# Using Network Analysis of Adolescent Self-Ratings of ADHD Symptoms to Identify Central Symptoms and Their Associations With Each Other and Global Functioning

**DOI:** 10.1177/00332941241308800

**Published:** 2024-12-11

**Authors:** Rapson Gomez, Stephen Houghton

**Affiliations:** 1458 School of Health and Biomedical Sciences, Federation University, Ballarat, VIC, Australia; Victoria University, Australia; Graduate School of Education, 2720 The University of Western Australia, Perth, WA, Australia

**Keywords:** ADHD, network analysis, adolescents, self-ratings, global functioning

## Abstract

This research examined the network properties (network graph, centrality, and edge weights) of the 18 ADHD symptoms, based on the self-ratings of 300 adolescents. The findings indicated the three symptoms with the highest centrality values were “inattention”, “wait”, and “interrupt”. For edge weights, there were positive large effect size associations for “lose” with “forgetful”, “fidget with “run”, “blurt” with “wait”, and “wait” with “interrupt”; and positive moderate effect size associations for “careless” with “instruction”, and “avoid” with “listen”. Five IA symptoms (“careless”, “instruction”, “avoid”, “distracted”, and “forgetful”) and one HI symptom (“quiet”) were associated negatively and significantly with global functioning. Overall, these associations and relations should be prioritized when planning treatment for ADHD. This is the first study to examine the network properties of ADHD symptoms for adolescent self-ratings. The implications of the findings for theory include a better understanding of the relationships and interrelations between ADHD symptoms, especially in terms of the clustering of IA and HI symptoms and their associations with global functioning. In practice, the findings indicate there are different symptoms that could be the focus for assessment and treatment according to the ADHD presentation type.

## Introduction

According to the fifth edition of the Diagnostic and Statistical Manual of Mental Disorders (DSM-5; American Psychiatric Association, 2013), Attention Deficit Hyperactivity Disorder (ADHD) is classified as a neurodevelopmental disorder, characterized by symptoms of inattention (IA), and hyperactivity/impulsivity (HI). Broadly, DSM-IV (APA, 1994) and DSM-IV TR, (2000) list these same 18 symptoms. Even though the latent variable framework has been the prevailing approach used for examining and understanding the structure of psychopathologies (including ADHD), a more recent framework, called the network approach (Borsboom, 2008; Borsboom & Cramer, 2013; Fried et al., 2017), has been proposed as an alternative approach. The latent variable framework provides a reflective view of psychopathology. That is, a psychological disorder is viewed in terms of a latent (unobservable) construct (i.e., the disorder in question) that causes several observable responses (i.e., the symptoms of the disorder). In contrast, the network framework proposes a formative view of psychopathology, in that it assumes the symptoms of a disorder are a causal system, interacting with each other in meaningful ways causing the disorder (Borsboom & Cramer, 2013). Although an increasing number of studies have started to use the network approach for examining ADHD symptoms (reviewed below), no research has yet used it for examining adolescent self-report ratings of ADHD symptoms. The present study sought to address this gap.

### Controversies on the Clinical Relevance of Adolescent Self-Reports

Obtaining the network properties of adolescent self-reports or self-ratings is only valuable if they are clinically useful. Relatedly, as there are data showing that adolescents rate their own ADHD behaviors at lower levels of severities compared to their parents and teachers, and that there is low to moderate levels of parent-adolescent and teacher- adolescent agreements for ADHD symptom reports (e.g., Hogue et al., 2014; Pelham et al., 2005; Schaughency et al., 1994; Smith et al., 2000), it has been suggested that adolescent self-reports are not relevant for ADHD diagnosis (of adolescents), and that only parent and teacher reports are sufficient for this purpose (Pelham et al., 2005). However, the logic underpinning arguments not favouring the use of adolescent self-reports appears flawed, as discussed below.

Achenbach (2011) highlighted that discrepancies across informant reports do not indicate that one source is more accurate than the other. Rather, they only show that they differ from each other. Consequently, we need to move from using such discrepancies as evidence that one source is more accurate than the other. This is especially relevant for ADHD because discrepancies between parent, teacher and adolescent ratings have also been explained in terms of situational specificity (Gomez & Gomez, 2015; Wolraich et al., 2004), i.e., poor agreement explained as reflecting actual differences in ADHD behaviours in different settings, such as parents observing home behaviours, and teachers observing school behaviours, and adolescents observing both home and school behaviours.

Notwithstanding the argument disfavouring the usefulness of adolescent self-reports, based on a review of self-report scales and best-practice guidelines for ADHD, Adler and Newcom (2011) called for the inclusion of self-report in diagnosis of ADHD in adolescents. This point has also been made by others (e.g., Becker et al., 2020; Brinkman et al., 2012; Bussing et al., 2011; Hope et al., 1999). In support of this practice, there are also studies showing that children and adolescents can provide reliable self-reports of their own ADHD symptoms (e.g., Bell et al., 2010; Klimkeit et al., 2006), and that there is high adolescent-parent agreement (Gomez & Gomez, 2015). Taking this into consideration, it can be argued that at this point, omission of adolescent self-reports for their diagnosis is premature. Instead, this should be considered as yet another valuable source of information that can contribute to more accuracy in their ADHD diagnosis. Consequently, acquiring the network properties of adolescent self-ratings of ADHD symptoms will be valuable.

### Network Analysis

Network analysis is used to empirically test a network model (Borsboom & Cramer, 2013; Boschloo et al., 2015). The variables in a network model are referred to as nodes, and the connections between nodes are referred to as edge weights. In the simplest network analysis, correlations (edges) between the nodes are computed. More often, partial correlations, controlling the relations between all other nodes, are computed. However, even in such instances, edge estimates can be inflated due to sampling error, and spurious edge estimates are produced (Epskamp et al., 2017). To overcome these problems, regularization can be used to estimate partial correlation networks (Epskamp et al., 2017). When the regularization involves Markov Random Fields (Epskamp et al., 2018), the network will show only the more important associations or edges (Borsboom & Cramer, 2013; von Klipstein et al., 2021), suppressing spurious edges to zero. Consequently, the overall associations found in such a network will not correspond to the associations from zero-order correlations, partial correlations, multiple regression analyses, and structural equation modelling (SEM; Epskamp et al., 2017).

In terms of output, a network analysis produces a network graph, centrality values, and edge weights. The network graph is a visualization of the network structure and is easy to interpret (Bringmann & Eronen, 2018), and the centrality value of a node reflects how closely it is connected to other nodes in the network. The commonly reported indices of centrality are betweenness (i.e., average distance of a node to all other nodes), closeness (i.e., the inverse sum of all the shortest paths), degree (also called strength, which is the number of non-zero edges), and expected influence (i.e., the sum of edge weights, accounting for both positive and negative edges) (see Opsahl et al., 2010; Robinaugh et al., 2016). For all these indices, nodes with higher centrality values are those more closely connected to other nodes.

### The Network Centrality Hypothesis

In this, the most central nodes are the most influential nodes in a network and they are the best intervention targets (Borsboom & Cramer, 2013; Robinaugh et al., 2020). Indeed, it has been suggested that treatment of the most central node, even those shown in cross-sectional network analyses, may result in the greatest overall treatment gains (McNally et al., 2015; Ruzzano et al., 2015). Relatedly, existing evidence shows that symptoms identified as highly central predict which nodes are more strongly associated with treatment change above and beyond other predictors (Rodebaugh et al., 2018). However, this view has been contested by some network analysis experts (Bringmann et al., 2019; Spiller et al., 2020), who pointed out centrality indices reflect the structure of the psychological network (i.e., the presence and strength of edges) and not the dynamics of the network (i.e., how symptoms influence each other’s presence), and that how the structure of a statistical network relates to causal influences of symptoms is unknown. Bringmann et al. (2019) and Spiller et al. (2020) concluded that while centrality indices may provide some information about which symptoms are more important for treatment, it is not straightforward. Generally, therefore, a network analysis of ADHD symptoms might reveal novel findings pertaining to the relative influence of the different symptoms and the associations between them, which might in turn enable more targeted interventions, and consequently better outcomes.

### Network Analyses of ADHD Symptoms

To date, several studies (e.g., Farhat et al., 2023; Goh et al., 2020; Lee et al., 2022; Liu et al., 2022; Martel et al., 2016; Preszler, 2020; Preszler et al., 2019; Silk et al., 2019; Waldren et al., 2024; Zhou et al., 2023) have reported the results from network analyses that have included only ADHD symptoms. Of these, three focused exclusively on ADHD symptoms obtained via rating scales (Liu et al., 2022; Martel et al., 2016; Preszler, 2020), while the other studies included other symptoms, such as sluggish cognitive tempo and Oppositional Defiant Disorder (ODD). In a network analysis, the set of variables entered as nodes will impact the network findings (Borsboom & Cramer, 2013; Boschloo et al., 2015). Thus, if the network includes other symptoms, the results will not reflect the “true” network properties of the ADHD symptoms. Consequently, the findings in the studies that focused exclusively on ADHD ratings (Liu et al., 2022; Martel et al., 2016; Preszler et al., 2019) are more relevant for the current study.

The current study focused on adolescent self-ratings, whereas previous research reporting the network properties of adolescents ADHD symptoms were based on parent ratings, adult ADHD symptoms, and self-ratings (Martel et al., 2016). Lee et al. (2022) reported the network properties of self-reported ADHD symptoms in emerging adults. In relation to parent ratings, Preszler (2020) reported that “inattention”, “seat” and “distracted” had relatively higher expected influence values than other symptoms, while Martel et al. (2016) reported that among the high centrality symptoms were “distracted”, “inattention”, and “follow”. For edge weights, there were close links for “talks” with the other impulsive symptoms; “inattention” with “fidget’; “run” with “seat”, and between “motor”, “fidget’ and “quiet”. In relation to parent ratings of children and adolescents together, Liu et al. (2022) reported that “distracted” and “fidgety” had relatively higher betweenness and closeness centrality values than the other symptoms, while “instruction” and “quiet” had relatively higher expected influence values than the other symptoms. Considering their network structure (see Figure 1(a) of Liu et al., 2022) strong edges included links between “loss” and “forget”; “avoid” and “instruction”, “careless” and “inattention”, “motor” and “quiet”, “interrupt” and “wait”, and “seat” and “run”. For parent ratings of only adolescents, Martel et al. (2016) reported that “distracted”, “inattention”, “forgetful” and “wait” were the top four centrality symptoms. For edge weights, they reported close links for “talk” and impulsive symptoms; “inattention” and “fidget”; and between “run”, “seat”, “motor”, “fidget” and “quiet”. For adult self-ratings, Martel et al. (2016) reported higher centrality for “distracted” and “inattention” than the other symptoms, and stronger edges between “inattention”, “avoid” and “distracted”; and between “motor”, “seat” and “fidget”. For self-ratings provided by emerging adults, Liu et al. (2022) reported that “fidget”, “distracted”, “inattention”, and “wait” had higher centrality values than the other symptoms. Considering their network structure, there were strong edges for “instruction” with “disorganized”, “forgetful” with “distracted”, “forgetful” with “lose”, “careless” with “inattention”, “motor” with “fidget”, “interrupt” with “blurts”, and “interrupt” with “wait”.

**Figure 1.**
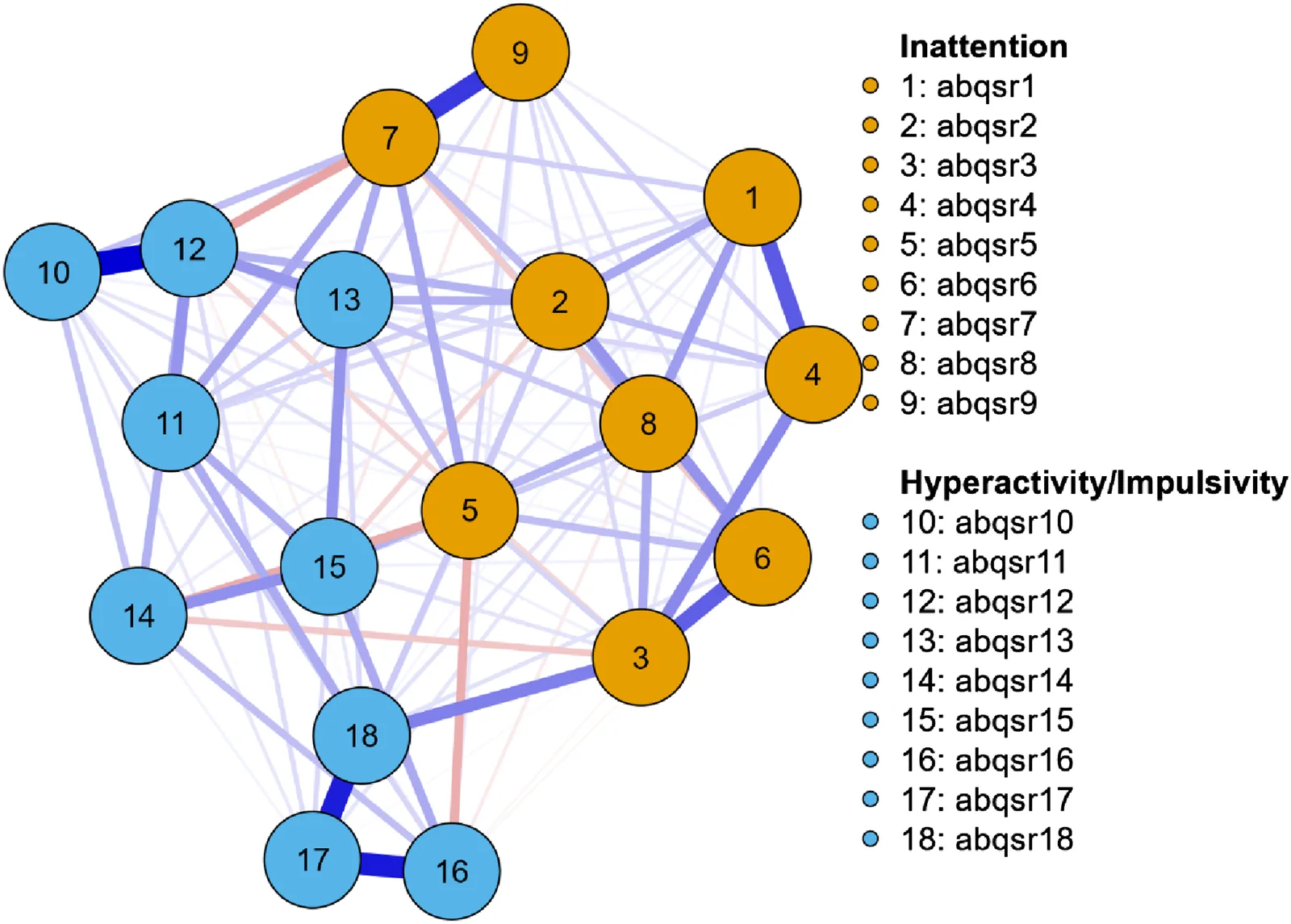
Network of the ADHD Symptoms. *Note*. Abqrsr refer to the ADHD symptoms in the A-DBRS-A, with the numbers indicating the symptoms in the order listed in DSM-5. Blue lines represent positive associations, and red lines negative associations. The thickness and brightness of an edge indicate the association strength. The layout is based on the Fruchterman-Reingold algorithm that places the nodes with stronger and/or more connections closer together and the most central nodes into the center. See Table 1 for brief descriptions of the nodes.

Overall, existing network findings for ADHD symptoms using network analysis reveal considerable variability. This may be related to the age groups examined, the informants and methods used for obtaining the ADHD symptom scores, and whether the studies focused exclusively on ADHD symptoms or included other variables. Martel et al. (2016) reported that ADHD symptom network structures became more differentiated over development, and that self-reports of ADHD symptoms in adulthood were dramatically different than other (mostly parent) reports of symptoms. Therefore, it can be speculated that adolescent self-reports most probably correspond to that reported by Lee et al. (2022) for emerging adult self-reports. Regardless, considering the potential value of adolescent network data for ADHD symptoms it can be argued that the absence of such data is a major limitation in understanding and managing ADHD in adolescents.

### Aims of the Current Study

The first aim of the current study was to conduct a network analysis for adolescent self-ratings of ADHD symptoms (network analysis 1). As highlighted earlier, although an increasing number of studies involving children and adolescents have reported network analysis properties of ADHD symptoms based on parent ratings and teacher ratings, no research has examined these properties for adolescent self-reported ratings. Considering the inclusion of adolescent self-report has been recommended for the diagnosis of ADHD (in adolescents) (Adler & Newcom, 2011), the findings from a network analysis will be valuable in providing important new information to what we already have from latent variable methods.

A second aim of the study was to extend the ADHD network model to examine how ADHD symptoms in the network were associated with global functioning (network analysis 2). Global functioning was a focus given it is included as axis V in the DSM-111 R and DSM-IV multiaxial diagnostic system and as such considered clinically meaningful. Regularized partial correlation was used in both network analyses. For the first network analysis, network graph, centrality, associations (edge weights) between the symptoms, and the stability of the findings for centrality and edge weights were examined. Since past studies have reported network analysis properties for self-ratings involving emerging adults (Lee et al., 2022), it was speculated that “fidget”, “distracted”, “inattention”, and “waiting” would be among the symptoms with higher centrality values, and that edges between “instruction” and “disorganized”, “forgetful” and “distracted”, “forgetful” and “lose”, “careless” and “inattention”, “motor” and “fidget”, “interrupt” and “blurts”, and “interrupt” and “wait” would be among the edges with stronger associations. For network analysis 2, it was expected that many of the ADHD symptoms would be associated negatively with global functioning.

In line with the ethics approvals granted by the institutional ethics committees the materials and de-identified data are available from the first author on request.

## Method

### Participants

A sample of 300 adolescents aged 12–17 years (females = 170, males = 130, mean age = 13.88 years [*SD* = 1.29]) provided ADHD ratings. These participants were also involved in previous publications (Gomez, 2012, 2013; Gomez & Gomez, 2015). However, these past studies examined gender invariance, mother-teacher-adolescent agreement, and item response theory properties, and were not related to network analysis. Greater detail about the participants can be found in these studies.

### Measures

#### Adapted Disruptive Behavior Rating Scale - Adolescent Version

The Adapted Disruptive Behavior Rating Scale - Adolescent Version (A-DBRS-A) used in the present study was adapted from the Disruptive Behavior Rating Scale (DBRS-P; Barkley & Murphy, 1998) to collect adolescent’s self-ratings of their ADHD symptoms. The nine DSM-IV ADHD IA symptoms and nine DSM-IV HI symptoms in the DBRS-P (Barkley & Murphy, 1998) were included in the A-DBRS-A. These symptoms are the same as in DSM-5 ADHD, but the word “often” was omitted in the list of symptoms. Participants were requested to: “Circle the number that best describes your behaviour over the past six months”. Like the parent version, each symptom was on rated on a 4-point scale (0 = never or rarely, 1 = sometimes, 2 = often, and 3 = very often). The wording of items in DBRS-P were amended for use in the adapted version to reflect a self-report format. Cronbach’s alphas for the ADHD (IA + HI), IA, and HI scales were .86, .82 and .89, respectively. In addition, all mothers of participants completed a demographic information questionnaire on their adolescents and family background.

#### Children’s Global Assessment Scale

The CAGS (Setterberg et al., 1992) was initially developed for clinicians to score a child’s lowest level of overall functioning during the past 6 months using a scale from 1 to 100. The parents of participants assign a single numeric score at any point on this scale, with higher scores indicating better global functioning. Respondents are asked to consider both behavioural and emotional functioning to account for functioning at home with the family, at school, with friends, and during leisure time. A simplified non-clinician version of this measure (the NC-CGAS) is available for completion by lay interviewers. The NC-CGAS has sound psychometric properties, including the ability to distinguish between children with and without emotional and behavioral disorders (Setterberg et al., 1992). The NC-CGAS is generally based on parent ratings, using the 1 to 100 scale. This measure has showed support for its reliability and validity for parent completion (Bird et al., 1996).

#### Procedure

Ethics approval (B01-03/536) for the research was provided by The Human Ethics Research Committee of the University of Ballarat, now renamed Federation University Australia. Directors of education, school principals, parents and classroom teachers also gave approval prior to the study commencing. Twenty-eight secondary schools were selected via stratified random sampling from all secondary schools in the State of Victoria (Australia). Of the 28 schools, 14 agreed to participate. Principals of these schools nominated potential classes and teachers of these classes who agreed to participate received sealed envelopes containing research material for forwarding to parents, via their students. Each envelope contained two sets of questionnaires (one for the mother and the other for the adolescent), with a return envelope. The set for mothers included a letter outlining the aims of the research, an invitation to participate and consent form, and the NC-CGAS. The set for adolescents also included the A-DBRS-A. Those who wished to be involved were asked to complete the consent form and to have their relevant child (ren) complete the A-DBRS-A. Information was also sought on the relevant adolescent’s age and gender, and the parent’s ethnicity, country of birth, highest education level completed, current employment status, and employment. The parents of participants also completed the NC-CGAS. Approximately 51% of the A-DBRS-A questionnaires were returned with fully completed adolescent ratings, and parent ratings of the NC-CGAS. These ratings were used in the current study. No compensation was given to anyone for participating.

#### Statistical Procedure

The initial network analysis focused on the 18 DSM-IV ADHD symptoms only. With 18 nodes in the network, the total number of estimated parameters was 190 [(19) + (18 × 19/2)] (Leme et al., 2020). The revised network analysis focused on the relationship of the 18 ADHD symptoms with global functioning. As there were 19 variables in the revised analysis, the number of estimated parameters was 210 [(20) + (19 × 20/2)]. With a sample size of 300 being more than the number of estimated parameters in both network models, the sample was deemed sufficient for both network analyses (Epskamp & Fried, 2018).

For both the initial and revised network analyses, the network module in Jeffreys’ Amazing Statistics Program (JASP) version 0.14.1.0 (JASP Team, 2018) was used. This module uses the R package for botnet (Epskamp et al., 2018) to conduct network analyses (Epskamp et al., 2012), and the qgraph to conduct network graphs. The module applies the least absolute shrinkage, together with the extended Bayesian Information Criterion (EBIC) model selection to produce regularized partial correlation networks (Foygel & Drton, 2010). With the gamma hyperparameter set at 0.5 it produces network models that are sparser and easier to interpret (Epskamp & Fried, 2018; Foygel & Drton, 2010), shows only the more important associations or edges (Borsboom & Cramer, 2013; von Klipstein et al., 2021), and suppresses spurious edges to zero.

Given the aims of the initial network analysis (i.e., focused on the network properties of only the 18 ADHD symptoms), our results for this network will focus on the network graph (data structure), centrality and edge weight values. The results related to establishing the stability and reliability of the centrality and edge weight findings are also reported. As our revised network model was aimed at examining the associations of the 18 ADHD symptoms with global functioning, the focus is only on edge weights. (Centrality is not relevant for this purpose).

Network graphs are produced in ways to make it easy to interpret. Specifically, nodes that are more similar are positioned closer to each other, and edge connections are colored so that positive associations are in blue and negative associations are in red. Additionally, stronger relationships have thicker and more denser lines. Fruchterman and Reingold (1991) was applied, which uses an algorithm to position the nodes, so that nodes with stronger correlations are placed near the center, and nodes with weaker correlations are positioned in the periphery.

The common reported indices of centrality are betweenness (i.e., the average distance of a node to all other nodes), closeness (i.e., the inverse sum of all the shortest paths), degree (also called strength, is the number of non-zero edges a particular node has), and expected influence (the sum of edge weights in weighted networks, accounting for both positive and negative edges, thereby providing an understanding of the cumulative influence a node has on a network ) (Opsahl et al., 2010; Robinaugh et al., 2016). For all indices, higher values indicated more centrality. Although all four centrality indices are reported, expected influence is used for evaluating the centrality of the nodes because it considers both positive and negative edges in the network, and therefore avoids the interpretative challenges found for the other centrality indices (Robinaugh et al., 2016). In a network, all edges that are present are significant (controlling for the other nodes in the network), whereas edges that are not significant will not be shown. For ease of interpretation of the edge weights, Christensen and Golino’s (2021), effect size guidelines were used (negligible ≤.14, small = ≥ .15 to <.25, moderate ≥.25 to <.35, and large ≥.35), with large and moderate effect sizes considered especially important.

When a network analysis is conducted, it is expected that the stability and reliability (i.e., likelihood that the network results will be replicated) of the centrality and edge findings are evaluated and reported. For the current study, this was evaluated for edge weights using bootstrap 95% non-parametric confidence intervals (CIs), with narrower CIs suggesting a more precise estimation of the edge (Epskamp et al., 2018). Large CIs for an estimated edge indicate stability of the edge weight. The stability of the centrality indices was evaluated using the case-dropping bootstrap (Epskamp & Fried, 2018), which examines if the correlation stability coefficients of the centrality indices remain stable after re-estimating the network with less cases. Generally, stability coefficients of 0.7 or higher are desired, although values of above 0.5 are considered acceptable (Epskamp et al., 2018). Both were estimated in the study with 1000 bootstraps.

In the second network model, the nodes included a measure for global functioning, in addition to the 18 ADHD symptoms. The primary focus in this model were the partial correlation (edges) for the ADHD symptoms with global functioning.

## Results

The occupational status of mostly father (or when father was not available - mother), based on the Australian Standard Classification of Occupations (ASCO; Australian Bureau of Statistics, 1997), indicated that participating adolescents were mainly from middle social class families. Ethnically, more than 95% reported a European background. Supplementary Table S1shows the descriptive statistics of the ADHD symptoms for these participants together. Although not shown, for all 18 ADHD symptoms in the A-DBRS-A, scores ranged from 0 to 3. The mean score was highest for HI “talk”, followed by IA “distracted”, and then HI “motor”. The symptom with the lowest mean score was IA “listen”.

### Missing Values and Descriptives

Only respondents with fully completed ADHD ratings were included. Thus, no missing values were evident in the data set. Supplementary Table S1 shows the mean and standard deviation scores for the 18 ADHD symptoms. In terms of mean scores, HI talk had the highest score, followed by IA distracted and then HI motor.

### Network Analysis 1

As there were 18 variables (nodes) in the network model, the number of potential edges was 153. However, the application of Markov Random Fields with regularization reduced the number of edges estimated in the network to 109, that is, sparsity = 0.29.

#### Visualization of the Network

The relationships of the ADHD nodes in the network are represented visually in Figure 1. As can be seen, the IA symptoms grouped together in one section of the network, and the HI symptoms were grouped together in another section of the network. The edges in the graph also showed varying lengths, intensities, thickness, and colours, thereby indicating much variation in strengths and directions of the association between the nodes.

#### Centrality of the Nodes in the Network

The centrality of the nodes in the network are shown in Table 1 (reproduced as a figure in Supplementary Figure S1). With reference to the expected influence values (used for evaluating centrality in this study), the three symptoms with the highest values were HI “wait”, IA “inattention”, and HI “interrupt”. Conversely, the lowest value was for HI “motor”.

**Table 1.** Centrality Indices of ADHD Symptoms From the Network Analysis.

Variable #/Brief description	Betweenness	Closeness	Strength	Expected influence
te01 – Careless	−0.94	−1.45	−0.53	0.51
te02 – Inattention	0.51	1.78	0.91	1.10
te03 - Listen	1.80	1.06	0.78	0.00
te04 – Instruction	−0.46	−0.80	−0.68	0.38
te05 - Disorganized	−0.62	0.10	0.44	−1.75
te06 –Avoid	−0.46	−0.09	−0.49	−0.42
te07 - Lose	1.48	0.95	1.73	0.19
te08 - Distracted	−0.62	−0.23	−0.12	0.55
te09 - Forgetful	−1.26	−1.81	−1.29	−0.48
te10 - Fidget	−1.26	−1.43	−0.94	0.15
te11 - Seat	−0.30	0.68	−0.75	0.31
te12 - Run	1.16	1.04	2.37	0.73
te13 – Quiet	−0.46	0.58	−0.57	0.47
te14 – Motor	−0.94	−0.74	−1.25	−2.36
te15 - Talk	0.19	0.39	0.60	0.00
te16 - Blurt	−0.13	−0.68	−0.58	−1.62
te17 - Wait	0.51	−0.15	0.31	1.25
te18 - Interrupt	1.80	0.80	0.05	1.00

*Note.* Higher numbers indicate that the variable is more central to the network; highest value for each symptom group is underlined within each index.

#### Edge Weights in the Network

The weight matrix between nodes is shown in Table 2, and as can be seen there were positive large effect size associations for IA “lose” with IA “forgetful”; HI “fidget” with HI “run”; HI “blurt” with HI “wait”; and HI “wait” with HI “interrupt”. Positive moderate effect size associations were evident for IA “careless” with IA “instruction”; IA “avoid” with IA “listen”. There were also positive small effect size associations for IA “careless” with IA “inattention” and IA “distracted”; IA “inattention” with IA “avoid” and IA “distracted”; IA “listen” with IA “instruction” and HI “interrupt”; IA “disorganized” with IA “lose”; IA “lose” with HI “seat”; HI “seat” with HI “run”; HI “run” with HI “quiet”; HI “quiet” with HI “talk”; and HI “motor” with HI “talk”. Positive negligible effect size associations were evident for 76 other edges (see Table 2), while for 11 other edges negative negligible effect size associations were evident (1 between IA symptoms, 1 between HI symptoms, and 9 between IA and HI symptoms). In this respect, “disorganized” had three negative associations, and “listen” and “avoid” had two negative associations. Overall, therefore out of a total of 190 (19 + 18 × 19/2) edges, there were only 94 (4 + 2 + 12+ 76) positive and significant edges (94/190 = 49.47%), with only 6 edges (3.16%) that could be considered as important. Additionally, 11 edges (5.79%) were not in the expected theoretical direction (negative and not positive).

**Table 2.** Weights Matrix Between the ADHD Symptoms From the Network Analysis.

Variable	1	2	3	4	5	6	7	8	9	10	11	12	13	14	15	16	17	18
1/Careless	.00	.15	.04	.27	.04	.03	.08	.16	.03	.01	.08	.00	.04	.03	.02	.00	.00	.01
2/Inattention		.00	.00	.10	.09	.18	.12	.15	.00	.00	.06	.13	.13	.00	−.07	.00	.09	.00
3/Listen			.00	.20	.07	.27	.00	.14	.06	.00	.03	−.07	.00	−.09	.05	−.01	.00	.21
4/Instruction				.00	.08	.00	.02	.03	.07	.02	.00	.00	.07	.00	.09	.00	.00	.00
5/Disorganized					.00	.11	.15	.01	.07	.05	.00	.00	.11	−.14	−.05	−.14	.06	.00
6/Avoid						.00	−.09	.09	.06	.04	.02	.02	.00	.00	.02	−.01	.01	.05
7/Lose							.00	.08	.33	.11	.15	−.15	.14	.00	.00	.00	.00	.00
8/Distracted								.00	.04	.00	.02	.09	.00	.00	.13	−.03	.04	.05
9/Forgetful									.00	.00	.00	.07	.00	.05	−.03	.04	.00	.00
10/Fidget										.00	.04	.42	.00	.10	.00	.00	.04	.08
11/Seat											.00	.18	.07	.00	.14	.03	.00	.14
12/Run												.00	.17	.13	.00	−.02	.06	.01
13/Quiet													.00	.00	.18	.00	.01	.07
14/Motor														.00	.18	.11	.02	.00
15/Talk															.00	.14	.06	.04
16/Blurt																.00	.38	.07
17/Wait																	.00	.38
18/Interrupt																		.00

### Stability of the Accuracy of Edge Weights and Centrality Strength Index

The results of the test used to evaluate the stability of the edges, estimated using bootstrap 95% non-parametric CIs, can be seen in Supplementary Figure S2. As shown, relatively small CIs around the estimated edge weights were evident, including zero, thereby supporting the stability of the edge findings in the study. When the case dropping bootstrapping method was applied to evaluate the stability of the centrality indices, the stability coefficient remained above .5 because the sample reduced to 25% of the original sample. This result indicated stability for the centrality indices in the study (see Supplementary Figure S3).

### Network Analysis 2

Table 3 shows the edge weights for the ADHD symptoms with global functioning (where higher scores indicate higher global functionality) included. As shown, five IA symptoms (“careless”, “instruction”, “avoid”, “distracted”, and “forgetful”) and one HI symptom (“quiet”) showed negative and significant associations with global impairment. Also, the HI symptom for “blurt” was associated positively and significantly with global impairment All significant associations were of negligible effect sizes.

**Table 3.** Edge Weights for the ADHD Symptoms With Academic Functioning and Global Functioning.

ADHD Variables	Global Functioning
1/Careless	−.10
2/Inattention	.00
3/Listen	.00
4/Instruction	−.12
5/Disorganized	.00
6/Avoid	−.07
7/Lose	.00
8/Distracted	−.02
9/Forgetful	−.04
10/Fidget	.00
11/Seat	.00
12/Run	.00
13/Quiet	−.04
14/Motor	.00
15/Talk	.00
16/Blurt	*.04*
17/Wait	.00
18/Interrupt	.00

*Note.* The numbers for the symptoms in column correspond to their positions for the diagnostic criteria in in DSM-IV.

## Discussion

The present study examined the network properties of adolescent self-rating of 18 ADHD symptoms. The network model produced indicated support for the stability (reliability) of the network findings for centrality and edge weights. For the network graph, the results demonstrated that although the set of IA symptoms and the set of HI symptoms were grouped together, these sets occupied different sections. The three symptoms with the highest centrality values (based on expected influence values) were: HI “wait”, IA “inattention”, and HI “interrupt”, while the lowest value was for HI “motor”. The edges with positive large effect sizes were: IA “lose” with IA “forgetful”; HI “fidget” with HI “run’; HI “blurt” with HI “wait”; and HI “wait” with HI “interrupt”; and the edges with moderate positive moderate effect sizes were IA “careless” with IA “instruction”, and IA “avoid” with IA “listen”. Overall, there were only 94 edges (49.47%) that were positive and significant. In addition, 11 edges (1 connecting IA symptoms, one connecting HI symptoms, and 9 connecting IA and HI symptoms), showed negative (and not the expected positive) connections. These involved “disorganized” (three negative associations), “listen” and “avoid” (both having two negative associations) and “inattention”, “lose”, “forgetful”, and “run” (one negative association each). In the network analysis that also included global functioning five IA symptoms (“careless”, “instruction”, “avoid”, “distracted”, and “forgetful”) and one HI symptom (“quiet”) had negative and significant associations with global impairment. All associations were of negligible effect sizes, however.

### Comparison of Findings in the Current and Past Studies

Based on the self-rating findings reported by Lee et al. (2022), we speculated that “fidget”, “distracted”, “inattention”, and “wait” would be among those symptoms with higher centrality values; and that edges between “instruction” and “disorganized”, “forgetful” and “distracted”, “forgetful” and “lose”, “careless” and “inattention”, “motor” and “fidget”, “interrupt” and blurt”, and “interrupt” and “wait” would be among the edges with stronger associations. Compared to Lee et al. (2022) the present findings also showed that “wait” and “interrupt” were among the symptoms with relatively higher centrality values, and that “fidget” with “motor”, and “wait” with “interrupt” were the associations with higher edge weights.

Martel et al. (2016) reported parent ratings specific to adolescents, and like this study the IA and HI symptom groups were in different clusters, with the HI symptoms not as closely associated with each other than the IA symptoms. In addition, “distracted”, “inattention”, “forgetful” and “wait” were the top four centrality symptoms. For edge weights, Martel et al. (2016) reported close links for “talk” and impulsive symptoms; “inattention” and “fidget”; and between “run”, “seat”, “motor”, “fidget” and “quiet”. In comparison, the current study found that “distracted”, “inattention”, “forgetful” and “wait” were the top four centrality symptoms; and relative strong edge weights were evident between “lose” and “forgetful”, “fidget” and “run”, “blurt” and “wait”, “wait” and “interrupt”, “careless” and “instruction”, and “avoid” and “listen”. A common finding in both studies was that “wait” and “inattention” were among the high centrality values symptoms.

### Implications of Network Findings

There are important theoretical and clinical implications, arising from this study. The present findings correspond more closely with the self-ratings of emerging adults in the Lee et al. (2022) study than the adolescent self-rating findings reported by Martel et al. (2016). This is in line with our initial speculations. Taken together, the present findings and those of Lee et al. (2022) suggest that for adolescent self-ratings, the more central symptoms are likely to be “wait” and “interrupt’, and possibly “fidget” and “distracted”. For edges, the stronger associations are likely to be between “fidget” and “motor”, and “wait” and “interrupt”, and possibly “instruction” and “disorganized”, “forgetful” and “distracted”, “forgetful” and “lose”, and “careless” and “inattention”.

Like past studies, the present findings also revealed that while the IA and HI symptoms were grouped together, these two groups were in separate clusters. This corresponds with a 2-factor (IA and HI) ADHD structure, as proposed in DSM-5 (APA, 2013) and demonstrated empirically in earlier research (e.g., Gomez et al., 1999). Related to this, little separation was evident between hyperactivity and impulsivity, therefore the separation of these symptom groups, as in ICD-10 (World Health Organisation, 1992), is not warranted.

The variability in the strengths and directions of the associations between the nodes suggested differential association between the ADHD symptoms. From a network perspective, nodes with high centrality are viewed as more important and influential in the model. As HI “wait”, IA “inattention”, and HI “interrupt” had the three highest centrality values, these symptoms are more central and could potentially be targets of assessment and intervention. This also means that for the treatment of those with the ADHD inattentive presentation, “inattention” is a major symptom for assessment and treatment. For the ADHD hyperactive/impulsive presentation, “wait” and “interrupt” are more central and could therefore potentially be the target of assessment and intervention. Also, for those with ADHD combined presentation, HI “wait”, IA “inattention”, and HI “interrupt” could be the focus of assessment and intervention.

In terms of edge weights, there were positive large effect size associations for IA “lose” with IA “forgetful”; HI “fidget” with HI “run”; HI “blurt” with HI “wait”; and HI “wait” with HI “interrupt”. Theoretically, these positive associations were expected. As our network analysis used regularized partial correlation (thereby showing only the more important associations between pairs of variables, controlling for all other variables in the model), we argue that our findings for edge weights provides a better understanding of the relationships and interrelations between the ADHD symptoms than that revealed from previous correlation analysis, multiple regression, and SEM studies of the ADHD symptoms (Borsboom & Cramer, 2013; von Klipstein et al., 2021).

Given the support for a two-factor oblique latent structure for ADHD symptoms (Gomez et al., 1999), our expectation was for only positive associations between all ADHD symptoms. However, as our findings indicated, only 94 edges (49.47%) were positive and significant. It could be argued therefore, that there was only a modest level of significant and positive associations between the ADHD symptoms, and even so, most were either of small or negligible effect sizes. Additionally, there were 11 edges that were negative and significant. Nine of these were IA and HI symptoms. Of these, “disorganized” had three negative associations, and “listen” and “avoid” had two negative associations. Together, these findings raise questions about the usefulness of the symptoms for disorganized, listen and avoid, and as such suggest the need for their revision.

As the findings in the network analysis that included global functioning showed that IA symptoms for “careless”, “instruction”, “avoid”, “distracted”, and “forgetful”; and one HI symptom for “quiet” were associated negatively and significantly with global functioning, these symptoms can be considered as relevant for ADHD. In contrast, as HI symptoms for “blurt” showed positive and significant associations with global functioning, this symptom has questionable relevance for ADHD, and their usefulness may need to be carefully reviewed in future taxonomies of ADHD.

### Study Limitations

Several limitations associated with the present study need to be acknowledged. First, despite the network approach assuming that the symptoms of a disorder is a causal system (Borsboom & Cramer, 2013), causality cannot be assumed in this present study because the data were cross-sectional. At best, the findings show spurious associations that can be eliminated for causal relations. Second, the sample of adolescents in this present study was nonclinical (i.e., recruited from the general Australian community) and therefore the findings are not directly generalizable to adolescents in clinical settings. Third, psychiatric comorbidities are the rule rather than the exception in ADHD and along with neurodevelopmental factors (Jogia et al., 2022) may significantly influence ADHD. This was not considered in the current study, and therefore the findings may have been confounded by these factors. Fourth, the data in the present study were self-ratings, and therefore the findings may not be applicable to data collected via clinical interviews. Fifth, further studies are required because only one sample was examined in the present study. Notwithstanding these limitations, the study reported is the first to examine the network properties of adolescent self-ratings of ADHD symptoms. Therefore, the novel insights offered have the potential to contribute to further understanding the diagnosis and treatment of ADHD in adolescents.

## Supplemental Material

Supplemental Material - Using Network Analysis of Adolescent Self-Ratings of ADHD Symptoms to Identify Central Symptoms and Their Associations With Each Other and Global Functioning Supplemental Material for Using Network Analysis of Adolescent Self-Ratings of ADHD Symptoms to Identify Central Symptoms and Their Associations With Each Other and Global Functioning by Rapson Gomez, Stephen Houghton in Psychological Reports.

## Data Availability

Data sharing not applicable to this article as no datasets were generated or analyzed during the current study.
