# Supplementary material for: Using Network Analysis of Adolescent Self-Ratings of ADHD Symptoms to Identify Central Symptoms and Their Associations With Each Other and Global Functioning
Source: Psychol Rep. 2024 Dec 11;129(5):3897–918. doi: 10.1177/00332941241308800 (PMC13530359; doi:10.1177/00332941241308800)
Supplement: Supplementary file 1 [file sj-pdf-1-prx-10.1177_00332941241308800.pdf]

Supplementary Table

Supplementary Table S1

Mean and Standard Deviation Scores for ADHD Symptoms

ADHD Variables	Mean	Standard Deviation
1/Careless	.870	.659
2/Inattention	.540	.714
3/Listen	.443	.684
4/Instruction	.590	.737
5/Disorganized	.580	.716
6/Avoid	.660	.721
7/Lose	.513	.672
8/Distracted	1.140	.889
9/Forgetful	.637	.770
10/Fidget	.767	.950
11/Seat	.507	.782
12/Run	.840	.858
13/Quiet	.533	.807
14/Motor	1.087	.960
15/Talk	1.230	.924
16/Blurt	.660	.808
17/Wait	.573	.739
18/Interrupt	.597	.768

Supplementary Figure S1

Centrality Plots (Betweenness, Closeness, Degree, and Expected Influence) in the Network for the Association in the Network of Each ADHD Node

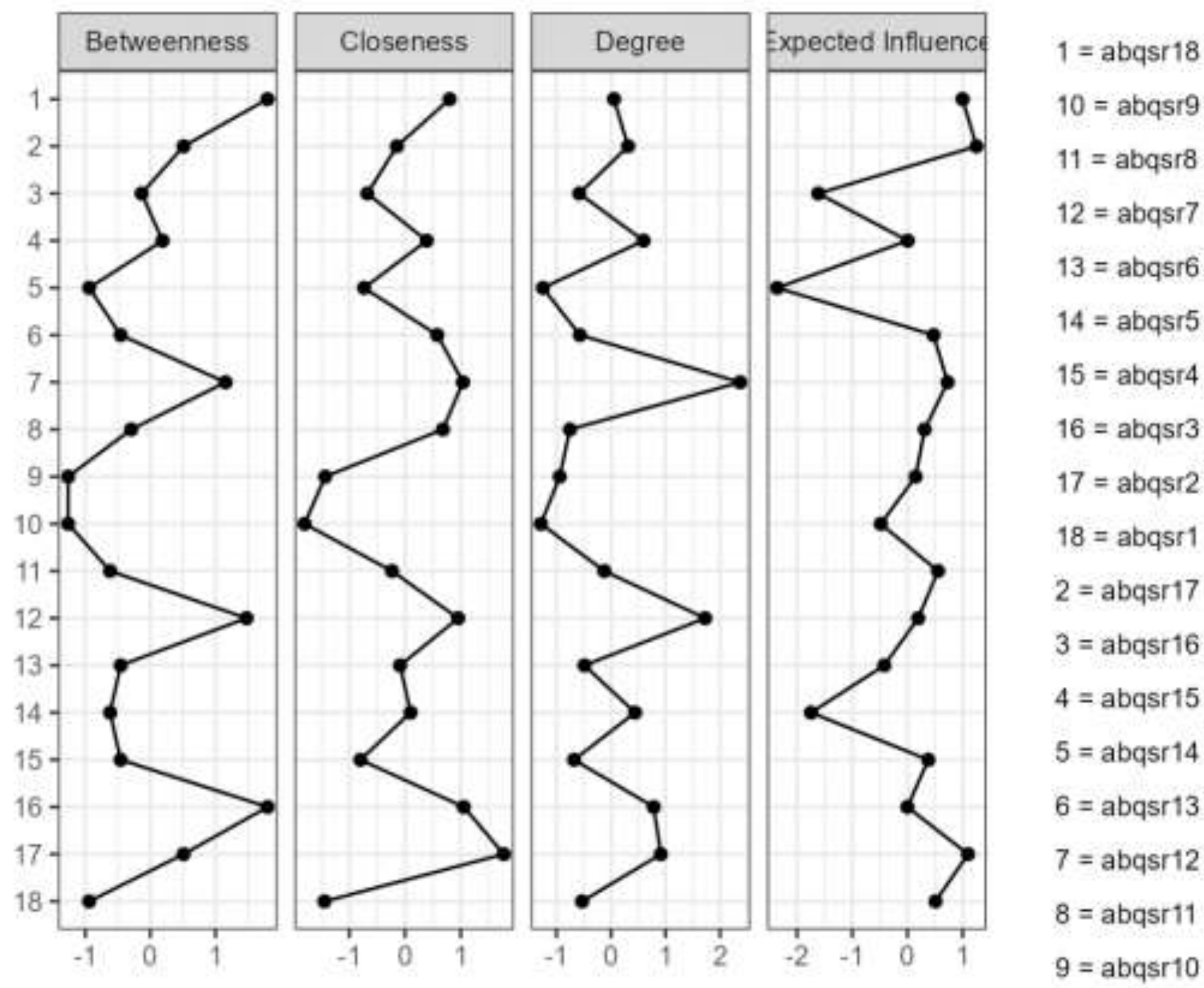

Note. Values shown on the x-axis are standardized z-scores. See Table 1 for brief descriptions of the nodes.

Supplementary Figure S2

Edge Stability Estimate for the ADHD Symptoms - Tested Using Non-Parametric Bootstrapped Estimate

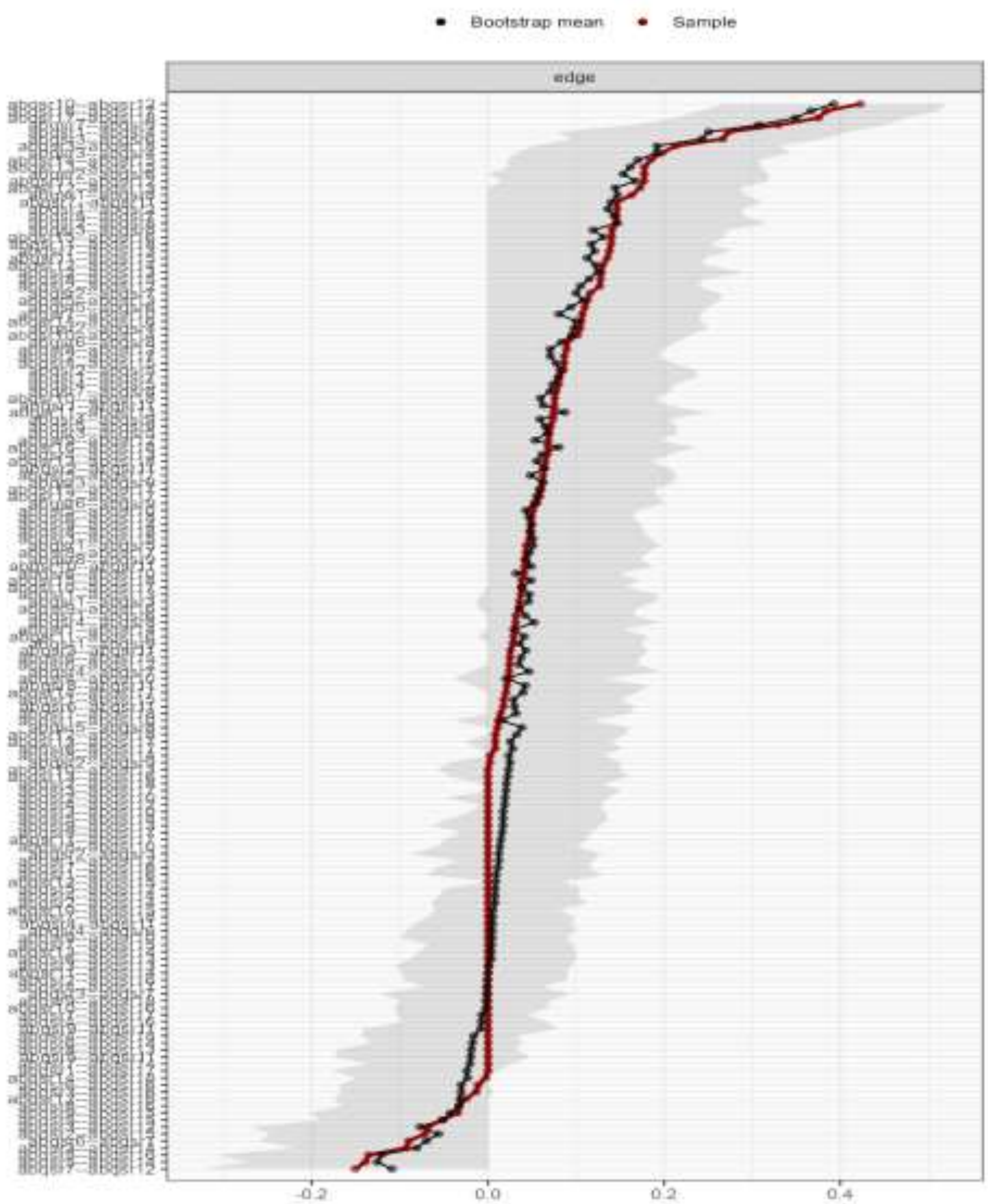

Note. The x-axis represents the edges, while every line on the y-axis represents a specific edge. The red line shows the estimate of the edge weights, and the gray bars the 95% confidence intervals for the estimates**.**

Supplementary Figure S3

Stability of Central Indices for the ADHD Symptoms

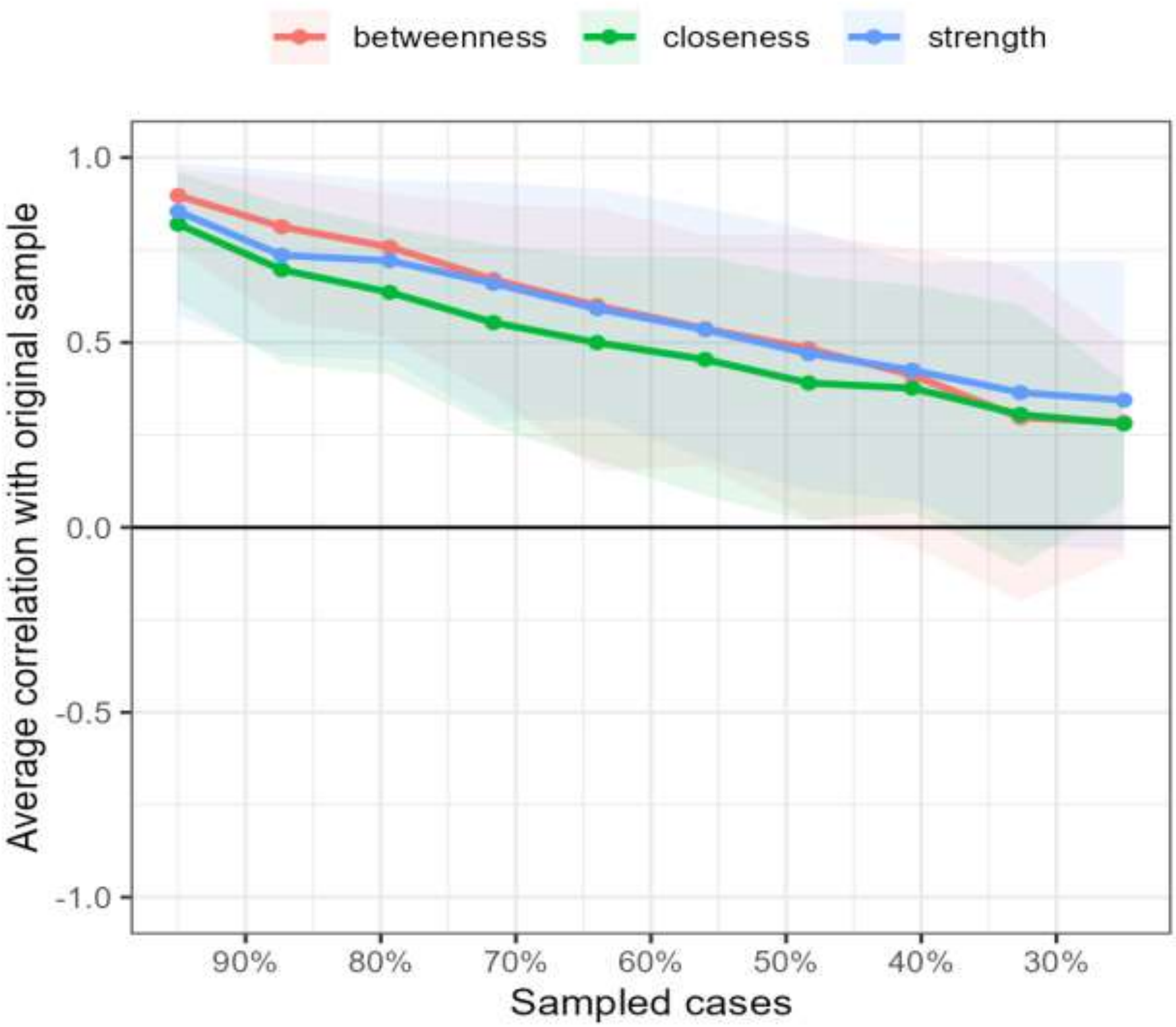

Note. The graph shows the average correlation between bootstrap centrality indices of networks sampled with node-dropping and network of the ADHD symptoms.
